# The Relationship between Persistent Organic Pollutants Exposure and Type 2 Diabetes among First Nations in Ontario and Manitoba, Canada: A Difference in Difference Analysis

**DOI:** 10.3390/ijerph15030539

**Published:** 2018-03-17

**Authors:** Lesya Marushka, Xuefeng Hu, Malek Batal, Tonio Sadik, Harold Schwartz, Amy Ing, Karen Fediuk, Constantine Tikhonov, Hing Man Chan

**Affiliations:** 1 Biology Department, University of Ottawa, 180 Gendron Hall, 30 Marie Curie, Ottawa, ON K1N 6N5, Canada; lmaru073@uottawa.ca (L.M.); Xuefeng.Hu@uottawa.ca (X.H.); 2 Nutrition Department, Faculty of Medicine, Université de Montréal, Pavillon Liliane de Stewart, 2405 Côte-Sainte-Catherine Street, Montreal, QC H3T 1A8, Canada; malek.batal@umontreal.ca (M.B.); amy.ingtam@gmail.com (A.I.); 3 Assembly of First Nations, 55 Metcalfe St #1600, Ottawa, ON K1P 6L5, Canada; tsadik@afn.ca; 4 Health Canada, Environmental Public Health Division, First Nations and Inuit Health Branch (FNIHB), Room 2000A Jeanne Mance Bldg. AL 1920A, Tunney’s Pasture, Ottawa, ON K1A 0K9, Canada; harold.schwartz@canada.ca (H.S.); constantine.tikhonov@canada.ca (C.T.); 5 Dietitian and Nutrition Researcher, Victoria, BC V8Y2V8, Canada; karen.fediuk@gmail.com

**Keywords:** persistent organic pollutants, type 2 diabetes, fish consumption, difference in difference model, long chain n-3 fatty acids, First Nations

## Abstract

We previously studied the association between fish consumption and prevalence of type 2 diabetes (T2D) in Manitoba and Ontario First Nations (FNs), Canada and found different results. In this study, we used a difference in difference model to analyze the data. Dietary and health data from the First Nations Food Nutrition and Environment Study, a cross-sectional study of 706 Manitoba and 1429 Ontario FNs were analyzed. The consumption of fish was estimated using a food frequency questionnaire. Fish samples were analyzed for dichloro diphenyldichloro ethylene (DDE) and polychlorinated biphenyls (PCBs) content. Difference in difference model results showed that persistent organic pollutant (POP) exposure was positively associated with T2D in a dose-response manner. Stronger positive associations were found among females (OR = 14.96 (3.72–60.11)) than in males (OR = 2.85 (1.14–8.04)). The breakpoints for DDE and PCB intake were 2.11 ng/kg/day and 1.47 ng/kg/day, respectively. Each further 1 ng/kg/day increase in DDE and PCB intake increased the risk of T2D with ORs 2.29 (1.26–4.17) and 1.44 (1.09–1.89), respectively. Our findings suggest that the balance of risk and benefits associated with fish consumption is highly dependent on the regional POP concentrations in fish.

## 1. Introduction

Type 2 diabetes (T2D) has become increasingly prevalent among Indigenous populations worldwide [1,2,3]. In Canada, the prevalence of T2D among First Nations is 3–5 times higher compared to the general population [4,5]. In addition, T2D has an earlier age of onset, is associated with greater micro- and macrovascular complications, and causes higher mortality among First Nations compared to the general Canadian population [4,5]. Lifestyle factors such as obesity, unhealthy diet, and lack of physical activity are well-recognized risk factors for T2D. However, other potential risk factors such as an exposure to environmental contaminants may also contribute to the high rates of T2D [6]. Epidemiological studies have confirmed positive associations between exposure to certain persistent organic pollutants (POPs) including polychlorinated biphenyls (PCBs) and dichlorodiphenyldichloroethylene (DDE), and T2D in general [7,8,9,10,11,12] and among Indigenous populations [13,14,15,16,17,18]. First Nations were reported to be exposed to higher levels of PCBs and DDE compared to the general Canadian population through traditional food, in particular, fish consumption [19]. On the other hand, traditional food provides significant nutritional benefits by contributing to the intake of essential nutrients including long chain omega-3 fatty acids (n-3 FAs) [20,21]. 

Fish consumption is widely promoted because of its beneficial health effects on cardiovascular diseases and mortality [22,23,24]. Recent evidence suggests that consumption of fish, rich in long-chain n-3 FAs (eicosapentaenoic acid (EPA), and docosahexaenoic acid (DHA)) may help prevent T2D since their improved lipid profile, reduces insulin resistance and inflammation [25,26]. Epidemiological studies reported contradicting results on the association between fish, n-3 FAs, and T2D. Some studies found inverse or protective associations [27,28,29], no association [30], or positive association between fish and n-3 FA intake, and T2D [31,32]. The discrepancy between the findings on the relationship between fish, n-3 FAs, and T2D may be possibly explained by differences in fish consumption patterns (n-3 FA content) as well as levels of contaminants present in fish [33]; however, these important factors were not considered in the previous studies. Wallin et al. found a statistically non-significant inverse association between fish consumption and T2D after adjustment for dietary PCBs and mercury exposure [34]. Turyk et al. reported that inverse associations between fish and blood glucose were stronger and statistically significant after adjustment for DDE exposure [35].

We previously reported differences in the association between fish consumption and the prevalence of T2D in First Nations living on reserve in Manitoba and Ontario, Canada. A negative dose–response relationship between the frequency of fish consumption and self-reported T2D was found in First Nations in Manitoba [36], whereas a positive association was observed in First Nations in Ontario [37]. The availability of traditional food species varies by ecozones and communities; however, the Manitoba and Ontario First Nations generally share similar cultural backgrounds and dietary preferences [38,39]. Demographic characteristics and other known risk factors were comparable between First Nations at the provincial level; however, significant differences in dietary POP exposure from fish consumption were found between Manitoba and Ontario. We hypothesized that the direction of the association was driven by dietary POP exposure. Due to the relatively higher intake of POPs from fish among Ontario First Nations than in Manitoba First Nations, it was thought that the adverse association of POPs may outweigh the protective associations of fish on T2D. Since dietary POPs were highly correlated with fish intake in the two groups of First Nations, it could be that regression analysis does not fully control and separate their individual effects. 

To test our hypothesis, we used a difference in difference (DID) model. The DID model is a statistical method widely used to evaluate the effectiveness of health care policy [40]. It allows the estimation of causal relationships between policy and outcome of interest using a series of observational studies [41]. The DID is considered a powerful method since it controls for unobserved background confounders that may influence the outcomes and thus allows for an assessment of the true impact of a predictor of interest [40]. The DID is also used in a cross-sectional setting [42,43]. This study aims to examine if dietary exposure to POPs may outweigh the benefits of fish on the prevalence of T2D, which helps to interpret our previous inconsistent findings in Manitoba and Ontario First Nations. Furthermore, we estimate the levels of dietary DDE and PCB exposure that increase the risk of T2D.

## 2. Methods

### 2.1. Manitoba and Ontario First Nations

**Figure 1:**
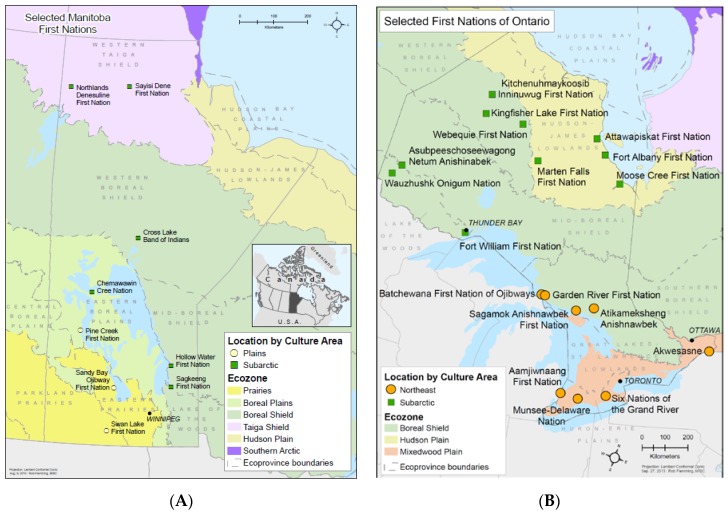
Map of participating First Nations communities in Manitoba (**A**) and Ontario (**B**) [38,39].

Data from the First Nations Food Nutrition and Environment Study (FNFNES) were analyzed. FNFNES is a cross-sectional study aimed to assess total diet and exposure to contaminants through traditional food consumption in First Nations adults living on reserves, south of the 60th parallel across Canada. Detailed information about the study design is available at www.fnfnes.ca. In brief, First Nations communities were randomly selected using a combined ecozone/cultural area framework to warrant that the diversity in ecozones and cultural areas were represented in the sampling strategy [38,39]. The sampling was completed in three stages: first, First Nations communities within each ecozone were randomly selected; second, within each selected community, 125 households were randomly sampled; and third, one adult in each household who was self-identified as being a First Nation person living on reserve aged 19 years and older was asked to participate in the study [38,39]. Estimation weights were calculated to obtain representative estimates of the total First Nations population. The current study combined data from First Nations in Ontario and Manitoba. Figure 1A,B show the geographic locations of the communities included in the survey. Participation rates were 82% in Manitoba and 79% in Ontario. Pregnant and breastfeeding women who reported having diabetes (n = 3) were excluded from the analyses in order to avoid potential misclassification of gestational diabetes. The total sample included 2132 participants (706 from Manitoba and 1426 from Ontario) aged 19 years and over. 

Ethics approvals were obtained from the Ethical Review Boards at Health Canada, the University of Northern British Columbia, the University of Ottawa, and the Université de Montreal. In addition, the Assembly of First Nations (AFN) Chiefs-in-Assembly passed resolutions in the support of this research. Participation in the study was voluntary. Written consent was obtained from each individual after an oral and written explanation of the project [38,39].

### 2.2. Data Collection

Household interviews were used to collect dietary data (24-h recall, a traditional food frequency questionnaire (FFQ)) and demographic characteristics (a socio, health, and lifestyle (SHL) questionnaire). The detailed information has been described previously [38,39]. The traditional FFQ consisted of 153 traditional food items in Manitoba and 150 in Ontario. Traditional food consumption was assessed over four seasons in the past year. The SHL Questionnaire included data on age, gender, weight, and height (measured or self-reported), physical activity, household size, education, and employment status and diagnosis of type 2 diabetes. All individuals were asked a question: Have you ever been told by a health care provider that you have diabetes? If participants responded “yes”, they were further asked about the type of diabetes and how long ago they had been diagnosed with diabetes.

### 2.3. Fish Sampling and Contaminants Analysis

Fish samples collected for contaminant analyses were representative of all fish species consumed by members in each community. Each community identified the most commonly consumed fish species and those that are of the most concern from an environmental perspective. The collected fish samples were analyzed for several POPs including total PCBs and DDE at Maxxam Analytics in Burnaby British Columbia and ALS Global, in Burlington, Ontario. 

### 2.4. Estimation of Fish, Dietary POPs (DDE, PCBs), and Long-Chain Omega-3 FA Intake

FFQ was used to estimate fish consumption. Daily fish intake (g/day) was calculated as follows: the total number of days over the past year when fish consumption was reported was multiplied by the age- and gender-specific portion size of fish species (g) reported through the 24-h recalls. To estimate total dietary PCBs and DDE intake, the amount of PCBs and DDE (nanograms/gram) in each fish species was multiplied by the total amount (grams) of each fish species consumed per day, summed up the amounts of PCBs and DDE from all fish species eaten per day, and divided by the body weight of each participant (ng/kg of body weight/day). Community-specific data of POP concentrations in fish species were applied to estimate PCBs and DDE intake for each participant. If no community-specific data were available, ecozone or regional contaminant data were used. 

Dietary assessments were validated through correlation analysis between mercury exposure from traditional food estimated using the FFQ and mercury concentrations in hair measured in First Nations. The correlation was statistically significant (Pearson correlation coefficient = 0.53).

The Canadian Nutrient File was used to estimate n-3 FA concentrations in fish species [44]. The n-3 FA concentrations were assumed to be the same for the same fish species in Ontario and Manitoba. For the purpose of the study, n-3 FAs means EPA + DHA from fish. The data are expressed as mg of EPA + DHA per gram of raw fish. 

### 2.5. Statistical Analyses

We use DID model to test our hypothesis. In the present study, the prevalence of T2D is the outcome of interest. Since a positive dose–response relationship between the frequency of fish consumption and self-reported T2D was previously found in Ontario First Nations, this cohort serves as the treatment group (exposed to POPs through fish consumption), whereas Manitoba First Nations serves as the comparison group (no/low exposure to POPs through fish) (Figure S1). The amount of fish consumption was used as a second source of difference. We explored a dose–response relationship by further separating fish consumers into two categories based on extent of intake (medium/high fish consumers). 

Preliminary analyses included the calculation of crude and standardized T2D prevalence, proportions for categorical variables, and means with standard deviations for continuous variables. The direct method was used to calculate the standardized prevalence of T2D, with the 2015 Canadian population as the standard population. For this analysis, fish consumption was divided into three categories: <5 g/day, 5–10 g/day, and >10 g/day. Logistic regression was performed using province, levels of fish intake and their interaction terms, with potential confounders as independent variables. This can be seen in Equation (1).

(1) $$Logit\left(outcome\right)=\alpha +\beta_{1}*ON+\beta_{2}*F_{M}+\beta_{3}*F_{H}+\beta_{4}*F_{MON}+\beta_{5}*F_{HON}+\gamma X+\varepsilon$$

In Equation (1), *α* is the intercept, the exponential form of *β* values are the odds ratios of each group, *γX* is a set of control variables, and ε is the model residual. Odds ratios (ORs) of having T2D were calculated for Ontario First Nations and fish consumption categories. The low fish consumer category (<5 g/d) served as a reference group. The beta (*β*) coefficients of main interest were *β*_4_ and *β*_5_. The *β*_1_ captures the difference in T2D prevalence between the Ontario and Manitoba First Nations, *β*_2_ and *β*_3_ capture the effect of fish consumption (n-3 FAs) on the prevalence of T2D, and *β*_4_ and *β*_5_, the interaction terms, capture the effect of POPs on the prevalence of T2D. Three underlying assumptions made in this study are (1) that n-3 FAs in fish decrease the risk of T2D; (2) that POPs from fish increase the risk of T2D, and (3) that the associations between fish (n-3 FAs) and POP exposure with T2D are similar in Ontario and Manitoba First Nations. 

Covariates were added into the model step by step to show the relative contribution of other risk factors and their influence on the magnitudes of *β*_4_ and *β*_5_. The control variables included in the final model were age, sex, body mass index (BMI), physical activity, total energy intake, education, and estimated intake of EPA + DHA, total PCBs, and total DDE. Age, BMI, total energy intake, education, and estimated intake of EPA + DHA, total PCBs, and total DDE were used as continuous variables. Physical inactivity and gender were used as dummy variables. The total sample size for regression analyses included 2080 participants (1326 females and 751 males) due to missing values for the control variables.

Segmented logistic regression was fitted to examine if the associations between dietary PCB/DDE intake and T2D prevalence changed at different doses. The adjusted ORs associated with each increase of 1 ng/kg/day in dietary PCB and DDE intake were reported. A forward procedure was adopted to show the relative contribution of other risk factors and their influence on the PCB/DDE effect size and the breakpoints. The final sets of covariates include age, sex, smoking, BMI, physical activity, education, total energy intake, and total fish intake. The proportion of missing data was less than 5%. All statistical analyses were performed using weighting variables in order to obtain representative estimates at the regional level. Results with a *p*-value of less than 0.05 were considered statistically significant. STATA statistical software, 14.2 (StataCorp, College Station, TX, USA) was used to perform statistical analyses. The segmented logistic regressions were performed with R (R Core Development Team). 

## 3. Results

The study population included 2132 First Nations participants (706 from Manitoba and 1426 from Ontario). Table 1 summarizes demographic characteristics of Ontario and Manitoba First Nations men and women. The crude prevalence of T2D was 22.9% in Ontario participants and 17.4% in Manitoba First Nations. After standardization to the 2015 Canadian population, the prevalence of T2D was higher among Manitoba participants (28.4%) compared to Ontario individuals (25%). The average age of the study sample was lower in Manitoba (42.3 years) compared to the Ontario sample (46.5 years). The mean BMI was comparable between men and women in both Manitoba and Ontario provinces, ranging from 29 to 31 kg/m^2^. Physical activity combines moderate and vigorous groups. In Ontario, adults tended to report more physical activity, a higher average total energy intake, and higher fruit and vegetable consumption than Manitoba adults. 

Table 2 presents the average consumption of the top five fish species and the concentrations of n-3 FAs and POPs in those fish. The most consumed fish species in both Manitoba and Ontario were walleye, lake whitefish, lake trout, northern pike and yellow perch. On average, they contributed 79% and 78% to the total fish intake in Manitoba and Ontario, respectively. Total fish consumption and total n-3 FA intake were higher among Ontario participants than in Manitoba individuals. Besides the top five consumed fish species, n-3 FA concentrations in other fish species were higher in Ontario. In regard to POP content, all selected fish species had higher concentrations of contaminants in Ontario than in Manitoba. In Ontario, DDE levels in the top five fish species ranged from 1.85 to 26.64 ng/g, and PCB levels ranged from 8.98 to 63.7 ng/g. In Manitoba, neither DDE nor PCBs were detected in walleye and yellow perch, but DDE levels ranged from 0.15 to 11.73 ng/g and PCB levels ranged from 0.03 to 9.24 ng/g in the rest of the fish species. The average concentrations of DDE and PCBs of all top five fish species were estimated to be 5 and 36 times higher in Ontario than in Manitoba, respectively. Lake whitefish and lake trout, compared with other commonly consumed fish species, contained the highest levels of n-3 FAs (1.24 and 0.73 g/100g of fish, respectively).

Table 3 summarizes total fish, dietary n-3 FAs, and POP intake by three categories of fish consumption (<5 g/d, 5–10 g/d, and >10 g/d) in First Nations men and women in Ontario and Manitoba. Men consumed more fish and omega-3 FAs that women did in both Ontario and Manitoba. Dietary exposure to DDE and PCBs, and n-3 FA intake was significantly higher in Ontario participants compared to Manitoba responders. 

**Figure 2:**
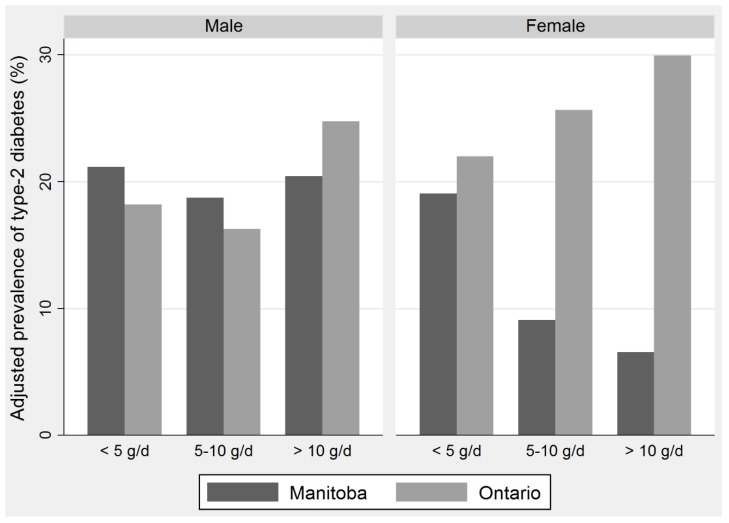
The prevalence of type 2 diabetes by categories of fish intake in Manitoba and Ontario First Nations males and females.

The associations between fish consumption, dietary POPs, and T2D prevalence are shown in Table 4. Model 1 shows the crude ORs, Model 2 was adjusted for age and gender, and Model 3 was further adjusted for BMI, physical activity, total energy intake, smoking, and education. Overall, the Ontario First Nations had a lower prevalence of T2D (OR = 0.53 (95% CI: 0.33–0.87) than the Manitoba Fist Nations. Medium and high consumption of fish was associated with lower T2D prevalence; however, the estimates were marginally or not statistically significant. The ORs of the two interaction terms reflect the association between POPs and the prevalence of T2D, after subtracting the association between fish intake (n-3 FAs) and T2D. Dietary POPs were positively associated with T2D. The magnitude of ORs became more prominent after additional adjustment for risk factors across models. In Ontario, the OR in the high fish consumers (3.53 (95% CI: 1.47–8.45)) was almost two times higher than that in the medium fish consumers (OR = 2.22 (95% CI: 0.86–5.68)). That translates into a nearly four-fold increase in the prevalence of T2D from low to high POP exposures. The magnitude of the association between POPs and T2D outweighed that between fish intake (n-3 FAs) and T2D (Figure 2). The association between frequency of fish consumption, dietary POPs, and T2D was also tested using fish consumption as a continuous variable (Table S1) and resulted in similar conclusions to analyses using categorical fish variables. 

Effect estimates were also examined in fully adjusted sex-stratified models (Table 4). A dose–response relationship for fish consumption was statistically significant in females, but not in males. In females, medium and high fish consumption showed statistically significant negative associations with T2D with OR = 0.29 (0.13–0.62) and OR = 0.16 (95% CI: 0.04–0.61), respectively. This translates into a nearly 80% decrease in the prevalence of T2D in high-fish-consumer, compared to low-fish-consumer, females. In males, the point estimate of ORs decreased from 1.45 to 0.99 from medium to high fish consumers but was not statistically significant. Dietary POP exposure was positively associated with the prevalence of T2D in both First Nations females and males. In females, the magnitude of the association in high fish consumers was about five times higher than that in medium fish consumers ((OR = 14.96 (95% CI: 3.72–60.11) and OR = 3.08 (95% CI: 1.13–8.42), respectively). This indicates that high exposure to dietary POPs resulted in a 15-fold increase in the prevalence of T2D compared to the low exposure to dietary POPs in First Nations females. In males, ORs increased from 1.79 (95% CI: 0.27–11.67) in the medium fish consumers to 2.85 (95%CI: 1.14–8.04) in the high fish consumers, in which the estimate was statistically significant. Thus, the effect of dietary POP exposure on the prevalence of T2D in males was lower than that in females (Figure 2). Gender differences in the association of dietary POPs with the prevalence of T2D in Ontario compared to Manitoba were examined using three-way interaction terms (sex, fish consumption, and location), which supported a stronger association of T2D with high vs. low fish consumers in Ontario for females compared with males (Table S2). Associations of T2D with medium vs. low fish consumers in Ontario did not differ significantly by sex.

The ORs of T2D associated with each 1 ng/kg/day in dietary PCB/DDE intake are shown in Table 5. Segmented logistic regressions with one breakpoint were fitted, and the identified breakpoints and slopes (i.e., ORs) before and after the breakpoints are shown for PCB and DDE separately. The breakpoint for DDE was around 2.11 ng/kg/day, before which, no significant increase in the prevalence of T2D was found, and after which, each 1 ng/kg/day increase in dietary PCB intake was associated with the OR = 2.29 (95% CI: 1.26–4.17) increase in the prevalence of T2D. The corresponding estimates for PCB were as follows: the breakpoint was 1.47 ng/kg/day, and each further 1 ng/kg/day increase was associated with the OR = 1.44 (95% CI: 1.09–1.89) increase in the prevalence of T2D. 

**Figure 3:**
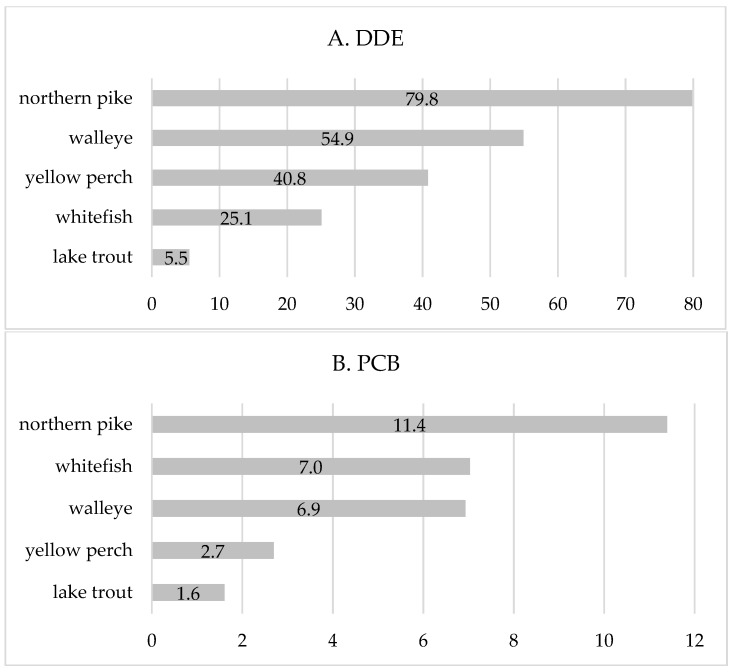
Amount of daily fish intake (g/day) with (**A**) DDE levels and (**B**) PCB levels below the estimated breakpoint in Ontario. DDE breakpoint = 2.11 ng/kg/day; PCB breakpoint = 1.47 ng/kg/day; reference body weight = 70 kg.

**Figure 4:**
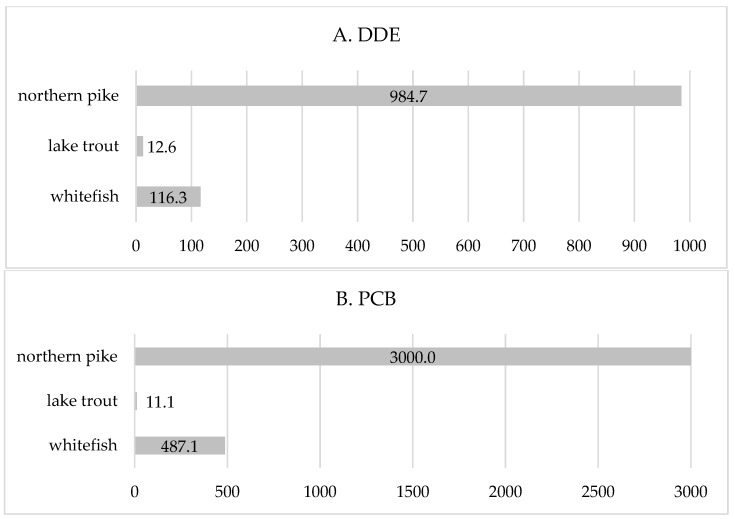
Amount of daily fish intake (g/d) with (**A**) DDE levels and (**B**) PCB levels below the estimated breakpoint in Manitoba. DDE breakpoint = 2.11 ng/kg/day; PCB breakpoint = 1.47 ng/kg/day; reference body weight = 70 kg.

Furthermore, we calculated the amounts of daily fish consumption (g/day) containing the concentrations of DDE and PCBs below the estimated breakpoints in Ontario and Manitoba separately (Figure 3 and Figure 4). The estimates are presented for the top five fish species, which constituted about 80% to the total fish intake. A body weight of 70 kg was used for the calculations. In Manitoba, only three fish species are presented since DDE/PCBs were not detected in two out of the five most consumed fish species (walleye and yellow perch) (Figure 4). These quantities of fish could be recommended as the maximum daily intake in order to prevent exceeding the DDE/PCB breakpoint exposure. The estimated amounts of daily intake of the top five fish species were lower in Ontario than in Manitoba due to significantly higher concentrations of DDE/PCBs in fish species. In Ontario, the estimated max daily fish consumption ranged from 5.5 (lake trout) to 79.8 g/day (northern pike) with respect to DDE exposure, and from 1.6 g/day (lake trout) to 11.4 ((northern pike) with respect to PCB exposure (Figure 3). In Manitoba, amounts of lake trout and whitefish that contain DDE/PCB breakpoint concentrations were 12.6 g/day and 116.3 g/day regarding DDE, and 11.1 g/day and 487.1 g/day regarding PCB exposure, respectively. In other fish species (northern pike, walleye, and yellow perch), the concentrations of DDE and PCBs were negligible or not detected. Overall, average daily consumptions of the top five fish species in Ontario and Manitoba First Nations were below the estimated amounts (Figure 3 and Figure 4).

The proportions of individuals with total DDE and PCB intake exceeding the estimated breakpoint exposure were 2.0% and 5.2% in Manitoba, and 9.7% and 27.9% in Ontario, respectively.

## 4. Discussion

Using the DID analysis, this study examined if relatively high POP exposure from fish may outweigh the protective associations of fish (n-3 FAs) on T2D in Ontario and Manitoba First Nations. Additionally, we examined the non-linear relationship between dietary PCBs and DDE exposure and T2D prevalence and estimated the threshold of daily dietary DDE and PCB exposure that increase the risk of T2D. The results show that dietary POPs were positively associated with the prevalence of T2D in First Nations living in Ontario. Stronger positive associations were observed among females compared to males. Higher fish (n-3 FAs) consumption was associated with a lower prevalence of T2D in Manitoba First Nations. When the data were stratified by gender analysis, statistically significant protective associations were found among females, but not in males. The breakpoints for DDE and PCB intake were 2.11 ng/kg/day and 1.47 ng/kg/day, respectively. Each further 1 ng/kg/day increase in dietary DDE/PCB intake increased the risk of T2D with OR = 2.29 (1.26–4.17) for DDE and OR = 1.44 (1.09–1.89) for PCBs, respectively. Based on these estimates, we calculated the approximate amount of fish consumption (by species) that could be recommended as maximum daily intake to prevent exceeding the DDE/PCB breakpoint exposure. 

Our findings on the positive relationships between POPs and T2D are consistent with a number of previous cross-sectional studies [10,11,13,15,35,42]. Lee et al. found a strong dose–response relationship between serum concentrations of six POPs, including DDE and PCBs, and T2D in a study among the US general population [45]. In a Native-American population, a significant association between diabetes and serum PCBs (OR = 3.29) and DDE (OR = 6.4) at the highest versus the lowest tertile was observed by [46]. Similar associations were reported by a study carried out among Inuit population [17] and First Nations in Canada [13]. Cross-sectional evidence on the relationship between serum POPs and T2D was also supported by prospective studies [11,47,48]. Former epidemiological studies investigated serum POP concentrations in relation to T2D prevalence, whereas we assessed dietary exposure to POPs via locally harvested fish intake. Since fish is considered the main source of exposure to contaminants among Aboriginal population [19], dietary POP intake from fish is a good indicator of exposure. Positive correlations between frequency of wild food consumption and serum POP levels were found in First Nations communities [19,49]. Fish consumption has been positively correlated with serum POP levels in other studies [11,13,50]. In the present study, traditionally harvested fish was estimated to be the main source of DDE and PCBs among all reported traditional foods [38,39].

In addition to epidemiological findings, experimental studies provide evidence of a causal relationship between POPs and insulin resistance [51]. Recent animal studies observed that chronic exposure to low doses of an environmentally relevant mixture of POPs via salmon oil consumption induced abdominal obesity, dyslipidemia, glucose intolerance, insulin resistance, and hepatic steatosis [52]. The in vitro experiment showed that treatment of differentiated adipocytes with nanomolar concentrations of POP mixtures impaired insulin-stimulated glucose uptake [52,53]. Several possible biological mechanisms have been proposed to explain the increased risk of T2D with exposure to POPs. Low-dose chronic exposure to POPs with endocrine-disrupting properties exhibits a diabetogenic effect through impairment of glucose and lipid regulations [54,55]. POPs may cause mitochondrial dysfunction via mutations in mitochondrial DNA and in nuclear genes, and through glutathione (GSH) depletion [56,57]. Mitochondrial dysfunction, in turn, plays a crucial role in chronic low-grade inflammation and may lead to ectopic fat accumulation in liver, muscle, and pancreas. Low-grade inflammation in adipose tissue is suggested to play an important role in the development of insulin resistance and T2D [56]. 

Several epidemiological studies reported that consumption of fish and n-3 FAs may prevent T2D [27,58,59], insulin resistance, glucose tolerance, and metabolic syndrome [60,61]. Nevertheless, systematic meta-analyses found geographical differences in the relationship between fish, dietary n-3 FA intake, and T2D [62]. Lee and Jacobs (2010) suggested that the direction of the associations between fish consumption and T2D may be driven by concentrations of beneficial nutrients (n-3 FAs) and harmful chemicals present in fish [33], which significantly vary by fish species and geographical location [63]. Additionally, POPs are known to be endocrine disrupting chemicals [64], with their hormonal effects starting to appear at low doses and diminishing when exposure increases [65]. On the other hand, the beneficial effect of fish (n-3 FAs) shows linear dose–response relations. Thus, the beneficial effects of fish on T2D may outweigh the harmful effects of POPs in the populations with relatively high fish consumption [66]. In contrast, in populations with low fish consumption, the beneficial effects of fish (n-3 FAs) might not be sufficient to outweigh the detrimental effects of POPs [66].

The balance of health benefits and potential risk of fish consumption have not been fully characterized. Several studies have quantified the risk and benefits of fish consumption to develop dietary recommendations. Many of those have focused on exposure to mercury only [67,68,69,70], while other studies have considered several POPs [71,72,73]. The relations between environmental chemicals and n-3 FAs with respect to coronary heart disease and cancer have been studied [74]. However, limited data are available on the risk and benefit associated with fish or n-3 FAs and POP exposure on T2D. Turyk et al. (2015) evaluated the joint effects of POPs and fish consumption on blood glucose in individuals with and without diabetes [35]. They found that consumption of total and saltwater fish was inversely associated with blood glucose, and the associations were more prominent after additional adjustment for DDE exposure. Additionally, Great Lake sport-caught fish (GLSCF) meals were inversely associated with blood glucose only after adjustment for DDE exposure, whereas positive associations of DDE and PCBs with blood glucose were strengthened after controlling for GLSCF meals. The authors emphasized the importance of adjusting for both fish intake and POP exposure in studies of populations consuming contaminated fish [35]. Christensen et al. (2016) examined cross-sectional associations between endocrine disorders (i.e., diabetes), fish consumption habits, and biomarkers among older male anglers in Wisconsin. The authors suggested that the effects of fish consumption on the risk for endocrine outcomes depend on the balance of the contaminants and nutrients [75]. In a population-based cohort study, Wallin et al. found no association between fish consumption and incidence of T2D. However, after additional adjustment for dietary PCB and mercury exposure, a statistically non-significant inverse association was observed between fish intake and the risk of T2D. The authors suggested that the beneficial effect of fish may be attenuated by the detrimental effect of POPs. Therefore, the net effect of fish consumption on T2D may depend on POP content in fish [34]. 

Gender differences in the relationship between certain POPs and diabetes were observed in other similar studies [7,48,76,77]. Rylander et al. found a strong positive association between diabetes and serum DDE levels in women only, whereas, in men, a positive association between PCB-153 and diabetes was observed [76]. In a prospective cohort study, serum PCB levels were positively associated with diabetes in women, but not in men [48]. In a study carried out in Anniston, Alabama, a city with a history of PCB manufacturing (1929–1971), adjusted ORs for the prevalence of diabetes in the highest versus the lowest quintile of serum PCBs was 2.8 in the total population, and 4.9 for women. No association was observed in men. Similarly, elevated serum DDE levels were associated with diabetes in women, but not in men [7]. The possible explanation of gender differences in the POP associations with diabetes may be differences in body fat composition. Women tend to have a higher proportion of body fat than men, with consequent greater accumulation and storage of lipophilic chemicals in adipose tissue. Additionally, several POPs are well-known endocrine-disrupting chemicals (EDCs) affecting the activity of estrogen, a hormone involved in the homeostasis of glucose and lipid metabolism [54]. Gender differences in the relationship between fish consumption and T2D have been observed: the authors of [59] found an inverse association between fish intake and T2D in women only.

The discrepancies in our findings reported in the previous studies in two groups of First Nations living in Manitoba and Ontario reflect differences in contaminant levels in fish species [36,37]. In fact, the concentrations of total PCBs and DDE in the most consumed fish species were estimated to be significantly higher in Ontario than in Manitoba. Consequently, dietary intake of PCBs and DDE in Ontario First Nations was much higher compared to Manitoba First Nations. Thus, elevated levels of POPs in fish diminish the beneficial effects of n-3 FAs on T2D. Significant variation in the levels of environmental contaminants in fish species between regions and within the same region were reported by other studies [69,78].

The average concentration of n-3 FAs in all fish species consumed by First Nations was higher in Ontario compared to Manitoba. Thus, there were background differences in n-3 FA intake between Ontario and Manitoba First Nations, with the higher n-3 FA intake estimated to be in the Ontario population. This may suggest that our estimates of the association between POPs and T2D may be underestimated. 

The protective effects of fish consumption on T2D were attributed to n-3 FAs based on evidence from epidemiological studies as well as our finding in Ontario and Manitoba First Nations. Besides n-3 FAs, fish contains other beneficial nutrients, such as high-quality protein, vitamin D, and selenium, which may also contribute to the protective effect of fish on T2D [79]; nevertheless, the results are still inconsistent [80,81]. Low levels of vitamin D were associated with greater insulin resistance, impaired beta-cell function, and a greater prevalence of metabolic syndrome in First Nations in Canada [82], whereas a study among Inuit in Greenland did not support a positive association between vitamin D levels and the risk of T2D [83]. 

There are several strengths of this study. First, the sample is large and representative of First Nations living on reserve across various ecological zones. Second, POP concentrations in locally harvested fish were measured in this study. The individual total dietary PCB and DDE intake was calculated based on community-specific data of POP content in fish species. Third, the difference in difference approach provides more strength in causal inference compared to other statistical methods when using observational study data. Finally, we found a strong dose–response relationship between dietary exposure to POPs, fish intake, and T2D.

This study has some limitations. First, the cross-sectional design of the study precludes us from asserting a causal relationship between POPs and T2D. In a cross-sectional setting, there is a risk of inverse causation. To examine if individuals diagnosed with T2D tend to change their diets and lifestyles, we performed sensitivity analyses. First, we compared the dietary intake and lifestyle habits between participants recently diagnosed with T2D (0–5 years) and individuals who had had T2D for a longer period of time (>5 years). The results showed that there were no statistically significant differences in dietary and lifestyle characteristics between the two groups in both Ontario and Manitoba First Nations [36,37]. Additionally, using data on self-reported dieting status, we examined whether dieting (i.e., limiting their caloric intake in order to lose weight) and non-dieting individuals with and without T2D differed by macronutrient intakes. This analysis found that macronutrient intakes were comparable between groups of First Nations in Manitoba and Ontario [36,37]. 

Second, given that data on the prevalence of T2D in the FNFNES were self-reported, we validated the data by comparing our estimates with those estimates reported by the First Nations Regional Health Survey, 2008–2010 (RHS) collected over the similar period of time [5]. The prevalence of diabetes in Manitoba and Ontario First Nations reported by the FNFNES was 22% and 24%, which was similar to the 21% and 21.6% reported by the RHS, respectively [36,37]. This evidence suggests that the prevalence rate of T2D reported in this study should be a reasonable estimate. 

Third, dietary POP exposure and n-3 FA intake were calculated from the same questionnaire information on fish intake, which can result in collinearity between variables. Dietary POP intake was estimated using community-specific data on POP content in fish species collected locally. The measured POP concentrations significantly vary between fish species and within species sampled from different regions. In contrast, only the n-3 FA concentration reported in the Canadian Nutrient File for each fish species was used for the estimation. Therefore, the risk of collinearity between POP with EPA + DHA and fish intake should be significantly decreased. 

Finally, there are limitations of the DID methods. First, the DID method assumes that, in the absence of the treatment (dietary POP exposure in this study), the average outcomes for the treated and control groups would have followed parallel trends. In this study, the corresponding assumption is that the associations between fish (n-3 FA) intake and T2D are similar in Manitoba and Ontario First Nations. However, we cannot test this assumption due to the cross-sectional nature of the survey. Second, the DID analysis requires the composition of population in the treatment and control groups before and after intervention (high vs. low fish intake in the current study) to be stable. We found that participants from Ontario and Manitoba were not the same in terms of age, gender, and the amounts and species of fish consumed, and we used multivariate regressions to adjust for the effects of these confounding factors.

## 5. Conclusions

Our findings suggest that relatively high dietary exposure to POPs such as PCBs and DDE may outweigh the beneficial associations between fish and T2D. This helps to explain the inconsistent findings between previous Ontario and Manitoba studies. Gender differences were found with stronger positive associations between dietary POP exposure and T2D prevalence among females. Furthermore, we were able to estimate the threshold of daily dietary DDE and PCB exposure that increase the risk of T2D. Potential risks or benefits associated with fish consumption were affected by regional differences in POP concentrations in traditionally harvested fish. Thus, dietary advice and guidelines should be tailored to reflect the regional differences. 

## Figures and Tables

**Table 1:** Descriptive characteristics of Ontario and Manitoba First Nations (FNs) participants.

Variables	Ontario			Manitoba		
	Total	Male	Female	Total	Male	Female
Sample size	1426	533	893	706	229	477
Type 2 diabetes	327	110	217	123	47	76
Type-2 diabetes weighted (%)	24.4	23.5	24.6	22.0	26.0	20.0
Type-2 diabetes standardized (%)	25.0	23.7	25.7	28.4	32.1	26.5
Age	46.5 (15.8)	47.3 (16.0)	45.9 (15.6)	42.3 (14.4)	43.1 (14.3)	42.0 (14.5)
BMI (kg/m^2^)	30.9 (5.9)	30.4 (5.4)	31.1(6.1)	30.3 (6.4)	29.0 (5.8)	30.9 (6.6)
Moderate to vigorous physical activity	498 (34.9)	241 (45.2)	257 (28.8)	189 (26.8)	85 (37.1)	104 (21.8)
Smoking (%)	723 (50.7)	276 (51.8)	447 (50.1)	444 (62.9)	136 (59.4)	308 (64.6)
Years of education	11.1 (3.8)	10.5 (3.5)	11.5 (3.9)	9.8 (2.5)	9.6 (2.7)	9.9 (2.4)
Total energy (kcal/day)	2042.1 (1026.8)	2344.5 (1222.1)	1861.6 (840.4)	1979.0 (1056.0)	2315.8 (1219.5)	1817.3 (926.5)
Fruit and vegetable intake (g/day)	157.6 (234.6)	141.7 (219.7)	167.1 (242.7)	113.1 (242.8)	88.8 (161.1)	124.8 (272.9)
Household size	3.4 (2.0)	3.0 (2.0)	3.6 (2.0)	4.4 (2.6)	3.9 (2.8)	4.6 (2.5)

Values are N (%) or mean (SD) unweighted; weighted estimates on type-2 diabetes prevalence represent provincial First Nations only and are not comparable between Manitoba and Ontario; type 2 diabetes standardized estimates represent prevalence, standardized to the 2015 Canadian population.

**Table 2:** EPA + DHA and persistent organic pollutant (POP) concentrations in the top five fish species in Ontario and Manitoba First Nations.

Fish Species	Ontario				Manitoba			
	Fish Intake	EPA + DHA	DDE	PCBs	Fish Intake	EPA + DHA	DDE	PCBs
	g/day	g/100 g	ng/g	ng/g	g/day	g/100 g	ng/g	ng/g
walleye	5.6 (13.5)	0.31 (0.05)	2.69 (3.36)	14.75 (19.44)	3.7 (9.1)	0.31 (0.05)	-	-
whitefish	2.5 (9.6)	1.24 (0.56)	5.89 (7.21)	14.56 (24.27)	2.0 (8.1)	1.24 (0.56)	1.28 (0.79)	0.21 (0.26)
lake trout	1.1 (5.6)	0.73 (0.14)	26.65 (24.32)	63.69 (83.54)	1.4 (5.9)	0.73 (0.14)	11.73 (5.76)	9.24 (2.58)
northern pike	1.7 (7.5)	0.27 (0.07)	1.85 (1.94)	8.98 (11.65)	1.0 (4.0)	0.27 (0.07)	0.15 (0.31)	0.03 (0.10)
yellow perch	0.5 (2.8)	0.25 (0.04)	3.11 (4.18)	33.18 (62.47)	0.2 (1.7)	0.25 (0.04)	-	-
subtotal	11.5 (28.0)	0.56 (0.42)	6.28 (11.82)	22.01 (40.49)	8.4 (18.4)	0.56 (0.42)	1.06 (2.92)	0.59 (2.21)
total	14.7 (34.1)	0.67 (0.48)	10.08 (19.62)	35.21 (68.06)	10.7 (24.5)	0.53 (0.28)	2.05 (4.37)	2.00 (5.40)

Values are mean (SD); “-”: not detected; EPA: eicosapentaenoic acid, DHA: docosahexaenoic acid, DDE: dichlorodiphenyldichloroethylene; PCBs: polychlorinated biphenyls; unweighted estimates of fish intake.

**Table 3:** Dietary EPA + DHA and POP intake by three categories of fish consumption.

Variables	<5 g/day		5–10 g/day		>10 g/day	
Ontario	Mean	95% CI	Mean	95% CI	Mean	95% CI
**Male**						
n	225		86		222	
Total fish intake (g/day)	1.28	0.93–1.64	7.11	6.58–7.63	62.19	41.48–82.89
EPA + DHA (mg/day)	22.04	12.50–31.59	119.87	90.14–149.60	935.09	636.61–1235.36
DDE (ng/kg/day)	0.08	0.04–0.12	0.31	0.13–0.50	3.19	1.60–4.76
PCBs (ng/kg/day)	0.37	0.25–0.49	1.41	0.69–2.12	11.28	7.20–15.37
**Female**						
n	573		113		207	
Total fish intake (g/day)	0.97	0.83–1.11	6.94	6.78–7.09	39.21	27.49–50.93
EPA + DHA (mg/day)	14.65	12.00–17.31	115.24	100.57–129.90	550.63	398.00–703.28
DDE (ng/kg/day)	0.06	0.04–0.09	0.5	0.26–0.65	3.61	1.39–5.84
PCBs (ng/kg/day)	0.249	0.17–0.32	1.723	1.05–2.39	9.86	4.47–15.24
**Manitoba**						
**Male**						
n	104		32		93	
Total fish intake (g/day)	1.61	0.66–2.57	6.9	6.29–7.50	34.4	19.74–49.07
EPA + DHA (mg/day)	6.27	2.58–9.97	28.6	21.45–35.74	195.4	72.73–318.07
DDE (ng/kg/day)	0.012	0.001–0.02	0.02	0.003–0.71	0.63	0.07–1.21
PCBs (ng/kg/day)	0.012	0.002–0.03	0.02	0.005–0.09	0.45	0.09–0.81
**Female**						
n	346		55		76	
Total fish intake (g/day)	1.31	1.08–1.54	7.02	6.75–7.29	30.85	26.92–34.78
EPA + DHA (mg/day)	5.22	4.00–6.44	31.71	27.65–35.76	183.12	144.41–221.85
DDE (ng/kg/day)	0.004	0.001–0.007	0.06	0.03–0.09	0.34	0.26–0.41
PCBs (ng/kg/day)	0.003	0.0004–0.006	0.07	0.003–0.14	0.26	0.17–0.35

EPA: eicosapentaenoic acid; DHA: docosahexaenoic acid; DDE: dichlorodiphenyldichloroethylene; PCBs: polychlorinated biphenyls; weighted estimates.

**Table 4:** Odds ratios (ORs) of the association between frequency of fish consumption and dietary POP exposure with type 2 diabetes prevalence (T2D) in Ontario compared to Manitoba First Nations.

Variables	Total Population			Female	Male
	Model 1	Model 2	Model 3	Model 3	Model 3
T2D in Ontario First Nations	0.53 ** (0.33–0.87)	0.52 ** (0.30–0.91)	0.53 * (0.27–1.03)	0.64 (0.29–1.44)	0.32 ** (0.12–0.82)
Medium fish consumers	0.43 ** (0.22–0.84)	0.58 * (0.31–1.09)	0.59 (0.29–1.18)	0.29 *** (0.13–0.62)	1.45 (0.46–4.56)
Medium fish consumers in Ontario	3.05 *** (1.32–7.08)	2.12 * (0.94–4.77)	2.22 * (0.86–5.68)	3.08 ** (1.13–8.42)	1.79 (0.27–11.67)
High fish consumers in Ontario	2.76 ** (1.25–6.09)	3.39 *** (1.49–7.68)	3.53 *** (1.47–8.45)	14.96 *** (372–60.11)	2.85 ** (1.14–8.04)
n	2080	2080	2080	1329	751

T2D: type 2 diabetes; low fish consumers: <5 g/day (reference group); medium fish consumers: 5–10 g/day; high fish consumers: >10 g/day; values are ORs (95% CI); Model 1: crude estimates; Model 2: adjusted for age and gender; Model 3: additionally adjusted for BMI, total energy intake, physical activity, smoking, and education; Ontario First Nations served as a treatment group and Manitoba First Nations served as a comparison (control) group; *** *p* < 0.01, ** *p* < 0.05, * *p* < 0.1.

**Table 5:** Segmented logistic regression of the association between dietary DDE/PCB exposure and T2D in Manitoba and Ontario First Nations *.

DDE Intake						PCB Intake					
Slope 1 (<BP)		BP		Slope 2 (>BP)		Slope 1 (<BP)		BP		Slope 2 (>BP)	
OR	95% CI	ng/kg/day	SE	OR	95% CI	OR	95% CI	ng/kg/day	SE	OR	95% CI
1.03	0.99–1.07	2.11	1.53	2.29	1.26–4.17	1.00	0.96–1.03	1.47	1.95	1.44	1.09–1.89

DDE: dichlorodiphenyldichloroethylene; PCBs: polychlorinated biphenyls; T2D: type 2 diabetes; BP: breakpoint; OR: odds ratio; CI: confidence interval; SE: standard error; OR measures the odds ratio of having T2D per 1 ng/kg/day change in DDE/PCB intake from fish; * Model was adjusted for age, gender, body mass index, smoking, physical activity, total energy, education, and total fish intake.

## Supplementary Materials

The following are available online at http://www.mdpi.com/1660-4601/15/3/539/s1, Figure S1: Dietary POP exposure (DDE+PCBs) by fish consumption categories in Ontario and Manitoba First Nations, Table S1: ORs of the association between fish consumption (continuous) and dietary POPs exposure and prevalence of type 2 diabetes in Ontario and Manitoba First Nations, Table S2: Gender differences of the association between frequency of fish consumption and dietary POPs exposure and prevalence of type 2 diabetes using 3-way interaction.

## Abbreviations

BMI	body mass index
CI	confident interval
DDE	dichlorodiphenyldichloroethylene
DID	difference in difference method
DHA	docosahexaenoic acid
EPA	eicosapentaenoic acid
FA	fatty acids
FFQ	food frequency questionnaire
FNFNES	First Nations Food Nutrition and Environment Study
OR	odds ratio
PCBs	polychlorinated biphenyls
POPs	persistent organic pollutants
SHL	Socio-health-lifestyle questionnaire
T2D	type 2 diabetes
