# Supplementary material for: The Relationship between Persistent Organic Pollutants Exposure and Type 2 Diabetes among First Nations in Ontario and Manitoba, Canada: A Difference in Difference Analysis
Source: Int J Environ Res Public Health. 2018 Mar 17;15(3):539. doi: 10.3390/ijerph15030539 (PMC5877084; doi:10.3390/ijerph15030539)
Supplement: Supplementary file 1 [file ijerph-15-00539-s001.pdf]

Supplemental materials

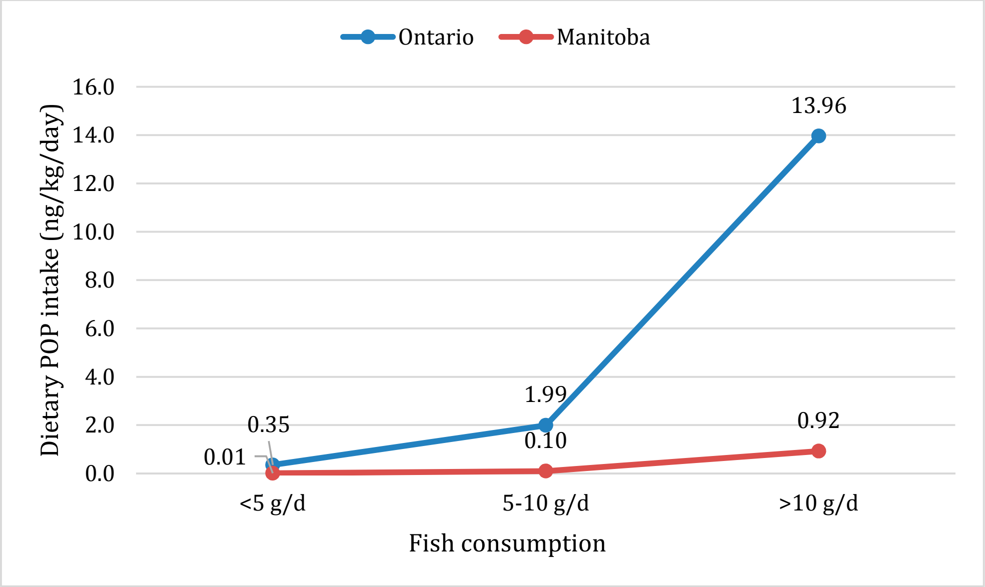

Figure S1. Dietary POP exposure (DDE+PCBs) by fish consumption categories in Ontario and Manitoba First Nations. (Dietary POPs intake from fish in Ontario (blue line) was significantly higher than in Manitoba (orange line) in all fish consumption groups. In the difference in difference model (DID), Ontario serves as a treatment group (exposed to a relatively high POP level through fish consumption) whereas Manitoba serves as the comparison group (no/very low exposure via fish comsumption)).

Table S1. ORs of the association between fish consumption (continuous) and dietary POPs exposure and prevalence of type 2 diabetes in Ontario and Manitoba First Nations.

Variables	Female	Male
	OR (95%CI)	OR (95%CI)
T2D in Ontario First Nations	0.65 (0.29–1.41)	0.68 (0.37–1.26)
Fish consumption (continuous)	0.93 ** (0.89–0.97)	1.00 (0.97–1.02)
Fish consumption in Ontario (continuous)	1.10 ** (1.05–1.15)	1.04 * (1.00–1.08)
n	1329	751

Model is adjusted for age, gender, BMI, total energy intake, physical activity, smoking, and education; Ontario First Nations served as a treatment group; Manitoba First Nations served as a comparison group; *** p<0.01, ** p<0.05, * p<0.1.

Table S2. Gender differences of the association between frequency of fish consumption and dietary POPs exposure and prevalence of type 2 diabetes using 3-way interaction.

Variables	OR (95% CI)
T2D in Ontario First Nations	0.38 ** (0.16–0.87)
Medium fish consumers	1.00 (0.53–1.87)
High fish consumers	0.89 (0.47–1.69)

Medium fish consumers in Ontario	1.33 * (0.31–5.81)
High fish consumers in Ontario	2.83 ** (1.00–7.27)
T2D in Ontario (female compared to male)	1.55 (0.57–4.21)
Medium fish consumers (female compared to male)	0.16 *** (0.06–0.45)
High fish consumers (female compared to male)	0.17 *** (0.05–0.54)
Medium fish consumers in Ontario (female compared to male)	2.20 (0.37–12.9)
High fish consumers in Ontario (female compared to male)	4.78 ** (1.11–20.52)

Low fish consumers: <5 g/d (reference group); medium fish consumers: 5-10g/d; high fish consumers: >10g/d; Model is adjusted for age, BMI, total energy intake, physical activity, smoking, education; Ontario First Nations served as a treatment group; Manitoba First Nations served as a comparison group; males is reference group for gender; *** p<0.01, ** p<0.05, * p<0.1.
